# Bioinformatics Approach for Prediction of Functional Coding/Noncoding Simple Polymorphisms (SNPs/Indels) in Human* BRAF* Gene

**DOI:** 10.1155/2016/2632917

**Published:** 2016-07-10

**Authors:** Mohamed M. Hassan, Shaza E. Omer, Rahma M. Khalf-allah, Razaz Y. Mustafa, Isra S. Ali, Sofia B. Mohamed

**Affiliations:** ^1^Faculty of Medical Laboratory Sciences, University of Medical Science and Technology, Khartoum, Sudan; ^2^Department of Biotechnology, Faculty of Applied and Industrial Science, University of Juba, Khartoum, Sudan; ^3^Faculty of Pharmacy, Omdurman Islamic University, Khartoum, Sudan; ^4^Faculty of Science, University of Khartoum, Khartoum, Sudan; ^5^Faculty of Medical Laboratory Sciences, Karary University, Khartoum, Sudan; ^6^Tropical Medicine Research Institute, Khartoum, Sudan

## Abstract

This study was carried out for* Homo sapiens* single variation (SNPs/Indels) in* BRAF* gene through coding/non-coding regions. Variants data was obtained from database of SNP even last update of November, 2015. Many bioinformatics tools were used to identify functional SNPs and indels in proteins functions, structures and expressions. Results shown, for coding polymorphisms, 111 SNPs predicted as highly damaging and six other were less. For UTRs, showed five SNPs and one indel were altered in micro RNAs binding sites (3′ UTR), furthermore nil SNP or indel have functional altered in transcription factor binding sites (5′ UTR). In addition for 5′/3′ splice sites, analysis showed that one SNP within 5′ splice site and one Indel in 3′ splice site showed potential alteration of splicing. In conclude these previous functional identified SNPs and indels could lead to gene alteration, which may be directly or indirectly contribute to the occurrence of many diseases.

## 1. Introduction

Genetic alterations (mutations) in general can be divided into two categories, inheritable (germline mutations) with 2% to 4% occurrence and sporadic (somatic mutations) [1, 2].* BRAF* coding gene, member of RAF family, located on chromosome seven (7q34), region from 140,715,951 to 140,924,764 base pairs which cover approximately 190 kb, is composed of 18 exons, and its translated protein name is “B-Raf proto-oncogene serine/threonine protein kinase.” This protein belongs to raf/mil family, which plays a role in regulating the MAP kinase/ERKs signaling pathway, which affects cell division, differentiation, and secretion [3]. Several studies reported the mutation prevalence in* BRAF* gene through various cancers, including non-Hodgkin lymphoma, colorectal cancer, malignant melanoma, thyroid carcinoma, non-small-cell lung carcinoma, and adenocarcinoma of lung [3–5]. Mutations in this gene have also been associated with various diseases such as cardiofaciocutaneous syndrome, a disease characterized by heart defects, mental retardation, and a distinctive facial appearance, Noonan syndrome, multiple lentigines syndrome or LEOPARD syndrome, giant congenital melanocytic nevus, and Erdheim-Chester disease [6, 7].

Single nucleotide polymorphisms (SNPs) markers are single-base changes in DNA sequence, with allele frequency of 1% or greater among population; it normally occurs throughout the genome with frequency of about one in every 1000 nucleotides, which is considered the simplest and common type of the genetic markers leading to DNA variation among individuals [8]. Nonsynonymous SNPs (nsSNPs) are one of coding SNPs types, important type of SNPs leading to the diversity of encoded human proteins, whereas they affect gene regulation by altering DNA and transcriptional binding factors, maintain the structural integrity of the cell, and affect proteins function in the different signal transduction pathways [9]. About 2% of the all known single nucleotide variants associated with genetic diseases are nonsynonymous SNPs and contribute to the functional diversity of the encoded proteins in the human population [10]. SNPs may be responsible for genetic diversity, evolution process, differences in traits, drugs response, and complex and common diseases such as diabetes, hypertension, and cancers. Therefore, identification and analysis of numerous SNP variations in genes may help in understanding their effects on genes product and their association with diseases and also could help in the development of new medical testing markers and individualized medication treatment [11].

1000 Genomes Project showed that most human genetic variation is represented by SNPs. Database of SNP (dbSNP) is one of the most databases serving as a central and public store for genetic variation since its initiation in September 1998 [12]. Any laboratory or individual can use the index variation, sequence information around polymorphism, and specific experimental conditions for further research applications. As with all NCBI resources, the data within dbSNP is available for free and in a variety of forms. In November 17, 2015, SNP database contained 160508575 number of* Homo sapiens* variants. From total number of variants, of which 144205811 were SNPs, 16064552 were Indels (single or multi-insertion/deletion). Database of SNP contains the results of HapMap and 1000 Genomes Projects (http://www.ncbi.nlm.nih.gov/snp/).

Through noncoding regions (3′ UTR, 5′ UTR), polymorphisms such as SNPs in microRNAs (miRNAs/mRNA) binding sites which are called mirSNPs can affect miRNAs function and then gene expression, resulting in many human diseases such as cancers [13]. Identification of SNPs responsible for phenotypes change is considered a difficulty, whereas it requires multiple testing for different SNPs in candidate genes [9]. One possible way to overcome this problem was to prioritize SNPs according to their structural and functional significance using different bioinformatics prediction tools. This study was focusing on functional SNPs within coding, 5′ UTR, 3′/5′ splice sites, transcription factor, and miRNA binding sites simple polymorphisms (SNPs/Indels) in human* BRAF* gene.

## 2. Materials and Methods

SNPs located in target gene were obtained from the database of SNPs (dbSNP); it is a public-domain archive for a broad collection of simple genetic polymorphisms. This collection of polymorphisms includes single-base nucleotide substitutions (SNPs), small-scale multibase deletions or insertions (also called deletion-insertion polymorphisms), and retroposable element insertions and microsatellite repeat variations (short tandem repeats or STRs) (http://www.ncbi.nlm.nih.gov/snp/). The related protein sequences are obtained from UniProt database (http://www.uniprot.org/).

SNP database contains SNPs or Indels within 3′/5′ UTR, 3′/5′ splice sites, coding synonymous, intron, and nonsynonymous which represent missense, nonsense, stop gain, and frameshift. In this study* Homo sapiens* SNPs and Indels (single insertion or deletion) within coding (nonsynonymous), 3′/5′ UTR, and 3′/5′ splice sites had been selected and submitted to bioinformatics tools for further investigation. Distributions of single variants are shown in Table 1.

About the main diagram of SNPs analysis, for missense SNPs, analysis was done by using three tools (SIFT server, PolyPhen, and SNAP2) and SNPs predicted as functional or damaging by previous triple servers were arranged in Table 2. More information about triple predicted SNPs is shown in Table 3. For frameshift SNPs, the analysis was done using SIFT server. By the same token for 3′ UTR SNPs and Indels, PolymiRTS database was used (Table 6). After that, for 5′ UTR SNPs (in transcription factor binding sites), PROMO tool was used (Table 7). Lastly for 3′/5′ splice sites SNPs and Indels, analysis was done using HSF tool (Table 8).

### 2.1. SIFT (Sorting Intolerant from Tolerant) Server

SIFT server is an online bioinformatics server that is used to predict the damaging effect of nucleotide substitution and frame shift (insertion/deletion) on protein function based on the maintenance degree of the amino acid residues in sequence alignments derived from closely related sequences with the main assumption; that is, evolutionarily conserved regions tend to be less tolerant to mutations, and so mutations in these regions mainly affect its function [14]. SIFT server has different input data order as follows: dbSNP reference number (rs ID number), protein sequence, and chromosome location. For this tool coding SNPs and Indels were separated from total and submitted as rs ID numbers for (missense, nonsense, and stop gain) SNPs and as chromosome location for frame shift Indels. SIFT server assigns score for each residue from 0 to 1, where ≤0.05 score is considered by the algorithm to be damaging amino acid substitutions and >0.05 score is predicting tolerance [15]. SIFT version 5.2.2 is available at http://sift.bii.a-star.edu.sg/index.html.

### 2.2. PolyPhen-2 (Polymorphism Phenotyping) Server

An online bioinformatics server automatically predicts the nsSNPs that affect with amino acid substitution structure and function of protein, using a comparative method. PolyPhen searches for protein 3D structures and make multiple alignments of homologous sequences and amino acid contact in several protein databases and calculate position-specific independent count scores (PSIC) for each of two variants and then computes the PSIC scores difference between two variants, where the higher PSIC score difference indicates that the functional impact of amino acid substitution is likely to occur [16]. PolyPhen-2 outcome can be one of the following: probably damaging, possibly damaging, or benign, with score range from 0 to 1 [9]. PolyPhen server is available at http://genetics.bwh.harvard.edu/pph2/index.shtml.

### 2.3. SNAP2 Server

SNAP2 is a trained classifier that is based on a machine learning device called “neural network.” It distinguishes between effect and neutral variants/nonsynonymous SNPs by taking a variety of sequence and variant features into account. The most important input signal for the prediction is the evolutionary information taken from an automatically generated multiple sequence alignment. Also structural features such as predicted secondary structure and solvent accessibility are considered. If available, also annotation (i.e., known functional residues, pattern, and regions) of the sequence or close homologs are pulled in. Predicting a score (ranges from −100 strong neutral prediction to +100 strong effect prediction), analysis suggests that the prediction score is to some extent correlated to the severity of effect [17] (https://rostlab.org/services/snap/).

From the total functional nsSNPs predicted by the three previous tools (SIFT server, PolyPhen, and SNAP2), the higher 15 functional nsSNPs (got higher predicted score) were selected for next analysis.

### 2.4. I-Mutant Suite

I-Mutant version 3.0 is a suite of support vector machine, based predictors integrated in a unique web server. It offers the opportunity to predict the protein stability changes upon single-site variations from the protein structure or sequence. I-Mutant result is designed as follows: DDG < 0: decrease stability, DDG > 0: increase stability, or DDG = 0: neutral [18]. I-Mutant 3.0 is available at http://gpcr2.biocomp.unibo.it/cgi/predictors/I-Mutant3.0/I-Mutant3.0.cgi.

### 2.5. CPH Models

A protein homology modeling prediction server, used to predict the 3D structure of proteins with an unknown 3D structure model, in CPH models the template recognition based on profile-profile alignment guided by secondary structure and exposure predictions [19]. Protein sequences requirements were submitted to CPH server to get the model as PDB file (for the structure that could not be predicted by automated Project HOPE server). The resultant PDB files were opened using Chimera program which was used to visualize the PDB structure (http://www.cbs.dtu.dk/services/CPHmodels/).

### 2.6. UCSF Chimera Model Software

**Figure 1:**
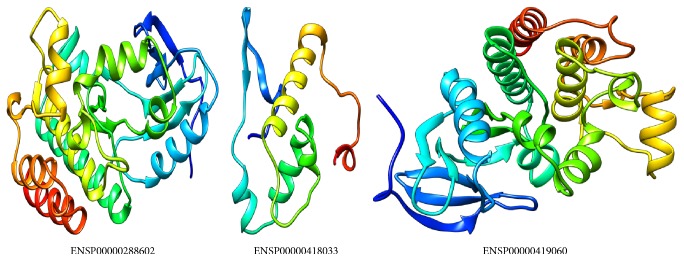
Showing proteins tertiary structure backbone and their protein secondary structures (alpha helix, beta sheet, and random coil) of higher deleterious nsSNP related proteins using CPH models 3.2 server and Chimera software. ID number below figures related to protein sequences records in UniProt database.

**Figure 2:**
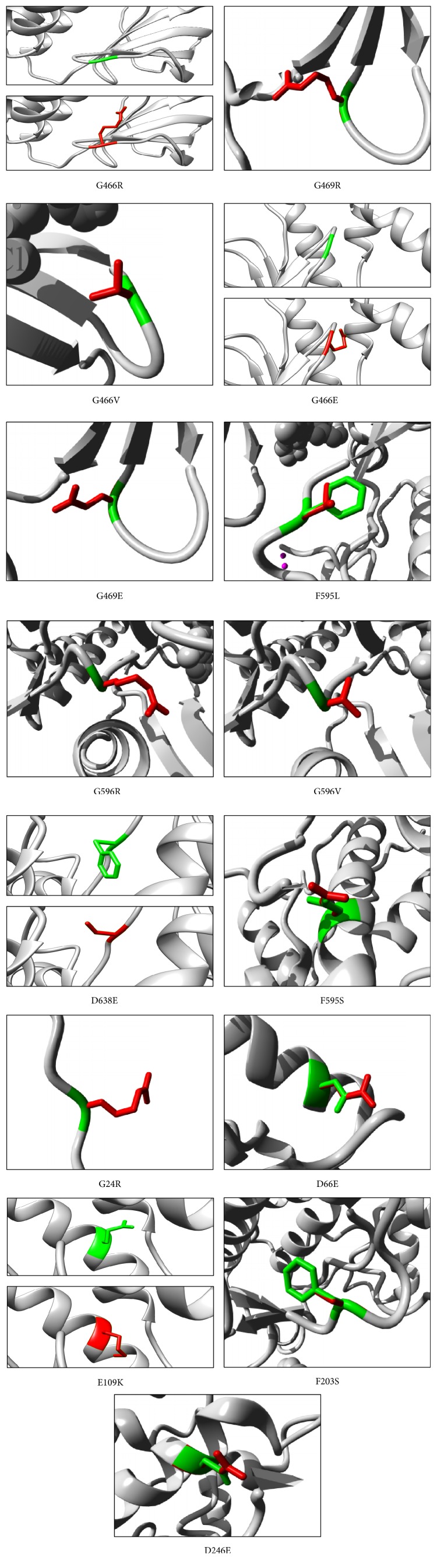
Close-up of the mutation. The protein is colored grey, and the side chain of both wild type (in green color) and mutant (in red color) residue shows in their target positions.

Chimera is a high-quality extensible molecular graphics program designed to maximize interactive visualization, analysis system, and related data [20]. This software was produced by University of California, San Francisco [9]. Chimera outcome was used to get high-quality images of, first, whole protein 3D structure that needed protein IDs ENSP00000288602, ENSP00000418033 and ENSP00000419060 (Figure 1) and, second, determined native and mutant residues for mutations that could not be detected by next automated Project HOPE server (Figure 2) (http://www.cgl.ucsf.edu/chimera/).

### 2.7. Automatic Protein Structural Analysis and Information Using HOPE Server

Automatic mutant analysis server can provide insight into the structural effects of a mutation. HOPE collects information from a wide range of information sources including calculations on the 3D coordinates of the protein by using WHAT IF Web services, sequence annotations from the UniProt database, and predictions by DAS services. Homology models are built with YASARA. Data is stored in a database and used in a decision scheme to identify the effects of a mutation on the protein's 3D structure and function. HOPE builds a report with text, figures, and animations that is easy to use and understandable for (bio)medical researchers [21] (http://www.cmbi.ru.nl/hope/method) (Figure 2).

### 2.8. PolymiRTS Database (3′ UTR)

It is an integrated platform for analyzing the functional impact of genetic polymorphisms (SNPs and Indels) within microRNAs binding sites [13]. Single variants within 3′ UTR were selected from total variants and submitted to PolymiRTS server, to check if these variants could disrupt or create new miRNA binding sites or have no impact at all. PolymiRTS is available at http://compbio.uthsc.edu/miRSNP/ (Table 6).

### 2.9. Effect of SNPs within 5′ UTR on Transcription Factor Binding Sites

PROMO is a virtual laboratory for the identification of putative transcription factor binding sites (TFBS) in DNA sequences from a species or groups of species of interest. TFBS defined in the TRANSFAC database are used to construct specific binding site weight matrices for TFBS prediction. The user can inspect the result of the search through a graphical interface and downloadable text files [22]. Input data was two sequences for each SNP within 5′ UTR: first sequence contained a wide nucleotide allele and the second contained a new substitution nucleotide as in Table 7 (http://alggen.lsi.upc.es/cgi-bin/promo_v3/promo/promoinit.cgi?dirDB=TF_8.3).

### 2.10. Effect of 3′/5′ Splice Sites SNPs/Indels (HSF Tool)

Human Splicing Finder (HSF) is a tool to predict the effects of mutations on splicing signals or to identify splicing motifs in any human sequence. It contains all available matrices for auxiliary sequence prediction as well as new ones for binding sites of the 9G8 and Tra2-*β* serine-arginine proteins and the hnRNP A1 ribonucleoprotein. It also developed new position weight matrices to assess the strength of 5′ and 3′ splice sites and branch points [23]. In this study HSF was used to detect the functional SNPs and Indels within 3′/5′ splice sites. Input data was nucleotide sequence containing the single substitution as SNP or insertion/deletion as Indel as in Table 8 (http://www.umd.be/HSF3/index.html).

## 3. Results and Discussion

Some information about total single variants and functional nsSNPs predicted with triple or double tools is obtained from many databases (dbSNP, UniProt, HapMap, 1000 Genomes Project, gene bank, and ClinVar) (Tables 1 and 2). In addition there was no functional SNP presented within HapMap or 1000 Genomes Project databases.

### 3.1. Predicted Results by SIFT, PolyPhen, and SNAP2 Servers

For 232 nsSNPs of* BRAF* gene, 111 variants were predicted to be damaging or effect by triple (SIFT, PolyPhen, and SNAP2) servers (Table 3). In addition one SNP (rs180177032, R70I) was predicted to be functional by double (SIFT and SNAP2) tools only. Furthermore five SNPs (V600M, L597V, L205V, V208M, and H2Q) were predicted as functional by double (SIFT and PolyPhen) servers only (Table 4). On the other hand, two Indels, frame shift (rs35546910, ch7:140834611; rs777474487, ch7:140783126-), showed no effect on protein at all.

From the previous results (Table 3), 15 nsSNPs with the maximum predicted score through triple servers were selected to predict their stability index (Table 5) and visualize wide and mutant residues in their protein 3D structure (Figure 2).

### 3.2. UTRs and Splice Sites

Results in untranslated regions showed lower number of functional SNPs and Indels than coding nsSNPs. 3′ UTR SNPs and Indels showed that five SNPs and one Indel were altered in microRNAs binding sites, which lead to disturbing or creating new binding sites (Table 6). Furthermore miRNAs associated with these functional SNPs/Indel are associated with many genes, and defect in these miRNAs could lead to effect on all associated genes expressions.

On the other hand, for 5′ UTR SNPs (five SNPs obtained), results showed that two SNPs were found in transcription factor binding sites with none being altered, and the remaining three were not located within any TF binding sites, meaning that none of five SNPs showed an effect on TF binding sites (Table 7). In addition, about the three single variants (two SNPs and one Indel) within 5′/3′ splice sites, analysis showed that one SNP within 5′ splice site and one Indel in 3′ splice site showed potential alteration of splicing (Table 8).

To date the complete mechanisms by which a nucleotide variant may result in a phenotypic change are for the most part unknown. In silico analysis using powerful software tools can facilitate predicting the phenotypic effect of nonsynonymous coding SNPs on the physicochemical properties of the concerned proteins. Such information is critical for genotype-phenotype correlations and also to understand disease biology. Given the fact that nsSNPs in critical cellular genes such as* BRAF* modify the normal programs of cell proliferation, differentiation, and death, they are believed to play an important role in disease predisposition. Therefore, efforts were made to identify SNPs that can modify the structure, function, and expression of the* BRAF* gene.

**Figure 3:**
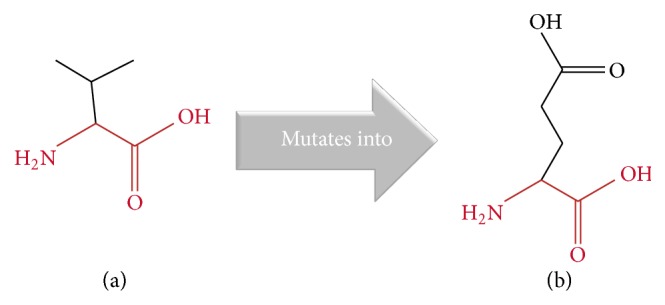
Showing the schematic structures of the original (a) and the mutant (b) amino acid. The backbone, which is the same for each amino acid, is colored red. The side chain, unique for each amino acid, is colored black.

Through one of the most significant* BRAF* mutations, when thymine is substituted with adenine at nucleotide 1799, it results in an amino acid substitution at position 600 from valine (V) to a glutamic acid (E), which is called V600E, located in the activation segment that has been found in many human cancers. For example, it was reported as the most common genetic mutation related to papillary thyroid cancer and occurs in approximately 45% of patients [24, 25]. In silico investigation also presented this mutation as highly damaging substitution that could cause a disease using SIFT and PolyPhen online tools. Furthermore Project HOPE server results showed that the wide type residue (V) is smaller in size (Figure 3), neutral in charge, and more hydrophobic. On the other hand mutant residue (E) is bigger in size (Figure 3), negatively charged, and less hydrophobic. In addition the mutated residue is located in a domain that is important for the activity of the protein and in contact with another domain that is also important for the activity. The interaction between these domains could be disturbed by the mutation, which might affect the function of the protein.

## 4. Conclusion

The current study shows the in silico analysis of genetic single variants within the coding region, 3′/5′ UTR and 3′/5′ splice sites of* BRAF* gene. These polymorphisms could directly or indirectly influence the intermolecular and intramolecular interactions of amino acid residues and protein expression and can culminate into disease risks. By analyzing the conformational changes and interactions of amino acid residues within* BRAF* proteins, we have identified significant structural and functional changes that can explain the activity deviations, caused by several mutations. Furthermore significant pathology or likely pathology showed association of many detected SNPs with many diseases through clinical variation database (http://www.ncbi.nlm.nih.gov/clinvar/). They include the following diseases: cardiofaciocutaneous syndrome, Noonan syndrome, LEOPARD syndrome, RASopathy, non-small-cell lung cancer, carcinoma of colon, adenocarcinoma of lung, thyroid cancer, malignant lymphoma, non-Hodgkin lymphoma. Screening for* BRAF* variants may be useful for molecular diagnosis and development of vital molecular inhibitors of genes pathways. This study demonstrates the significance of different bioinformatics tools to figure out the phenotypic changes and protein function, associated with the structure-function relationship of* BRAF* gene. More evidence is required for the involvement of deregulated miRNA networks in cancer development. Resultant SNPs can be applied for further investigation and diagnosis of many associated diseases.

## Figures and Tables

**Table 1:** Distributions of SNPs and Indels.

Regions	Number of SNPs	Number of Indels	Total
All over* BRAF* gene	9585	1111	10696
Coding	350	2	352
(i) Missense	228	—	228
(ii) Nonsense and stop gain	4	—	4
(iii) Frame shift	—	2	2
3′ UTR	331	31	362
5′ UTR	5	—	5
3′ splice site	1	1	2
5′ splice site	1	0	1

**Table 2:** Gene, protein, and clinically significant information of triple damaging predicted SNPs.

SNP ID	Ch7 location	Nucleotide change	Protein ID	Amino acid change	Clin/sig
rs150050723	140534634	T|G	ENSP00000288602	Q93H	—
rs180177034	140501336	C|G	ENSP00000288602	A246P	Path/3
rs387906660	140501350	G|A	ENSP00000288602	T241M	Path/3
rs387906660		G|C	ENSP00000288602	T241R	
rs387906661	140501351	T|G	ENSP00000288602	T241P	Path/5
rs397507466	140501337	T|G	ENSP00000288602	L245F	Path/2
rs397507467	140501332	A|G	ENSP00000288602	F247S	L.Path/1
rs397509343	140501331	A|C	ENSP00000288602	F247L	L.Path/1
rs267601317	140494148	G|A	ENSP00000288602	P367L	—
rs199927105	140482930	G|T	ENSP00000288602	P402H	—
rs121913348	140481417	C|G	ENSP00000288602	G464A	Path/3
rs121913348		C|A	ENSP00000288602	G464V	L.Path/1
rs121913348		C|T	ENSP00000288602	G464E	
rs121913353	140481412	C|G	ENSP00000288602	G466R	L.Path/1
rs121913357	140481403	C|T	ENSP00000288602	G469R	—
rs121913376	140481397	C|A	ENSP00000288602	V471F	Path/1
rs121913376		C|T	ENSP00000288602	V471I	L.Path/1
rs180177033	140481420	A|C	ENSP00000288602	I463S	Path/1
rs397507473	140481405	A|G	ENSP00000288602	F468S	Path/2
rs121913349	140481418	C|G	ENSP00000288602	G464R	—
rs121913371	140481478	G|A	ENSP00000288602	R444W	—
rs121913351	140481411	C|G	ENSP00000288602	G466A	Path/2
rs121913351		C|A	ENSP00000288602	G466V	
rs121913351		C|T	ENSP00000288602	G466E	
rs121913355	140481402	C|G	ENSP00000288602	G469A	Path/1
rs121913355		C|A	ENSP00000288602	G469V	
rs121913355		C|T	ENSP00000288602	G469E	
rs180177036	140477853	C|G	ENSP00000288602	L485F	Path/2
rs180177038		C|T	ENSP00000288602	E501K	
rs180177039		T|C	ENSP00000288602	E501G	
rs397507474	140477861	T|G	ENSP00000288602	K483Q	Path/2
rs397507475	140477854	A|G	ENSP00000288602	L485S	Path/2
rs397507476	140477811	T|G	ENSP00000288602	K499N	Path/1
rs397507477	140477795	G|A	ENSP00000288602	L505F	L.Path/1
rs375520366	140476806	G|A	ENSP00000288602	P490S	Path/1
rs180177041	140476806	C|G	ENSP00000288602	G534R	Path/1
rs397507479	140476811	C|T	ENSP00000288602	C532Y	Path/1
rs180177040	140453987	T|G	ENSP00000288602	N581H	Path/2
rs180177040		T|C	ENSP00000288602	N581D	
rs397507481	140454006	G|C	ENSP00000288602	H574Q	Path/1
rs121913341	140453150	A|C	ENSP00000288602	F595L	Path/2
rs121913361	140453149	C|G	ENSP00000288602	G596R	Un.S/1
rs121913364	140453134	T|C	ENSP00000288602	K601E	—
rs121913366	140453145	A|T	ENSP00000288602	L597Q	Path/1
rs121913366		A|C	ENSP00000288602	L597R	
rs121913370	140453193	T|C	ENSP00000288602	N581S	L.Path/1
rs121913375	140453139	G|A	ENSP00000288602	T599I	Path/1
rs397507483	140453148	C|A	ENSP00000288602	G596V	Path/3
rs397507484	140453133	T|A	ENSP00000288602	K601I	Path/1 L.Path/1
rs121913225	140453151	A|G	ENSP00000288602	F595S	—
rs121913337	140453153	A|T	ENSP00000288602	D594E	—
rs121913362	140453159	T|C	ENSP00000288602	I592M	—
rs121913365	140453132	T|G	ENSP00000288602	K601N	—
rs372569965	140453127	C|T	ENSP00000288602	R603Q	—
rs180177042	140449165	A|T	ENSP00000288602	D638E	Path/2
rs397507485	140439727	C|T	ENSP00000288602	R671Q	Path/1
rs397507486	140439613	T|C	ENSP00000288602	Q709R	Path/1
rs55715359	140439664	A|G	ENSP00000288602	L692S	—
rs397507487	140434543	G|A	ENSP00000288602	R719C	Path/1
rs200490285	140434452	G|T	ENSP00000288602	A749D	—
rs368528867	140434542	C|A	ENSP00000288602	R719L	—
rs180177040	140453987	T|G	ENSP00000418033	N9H	Path/2
rs121913341	140453150	A|C	ENSP00000418033	F23L	Path/1 L.Path/2
rs121913361	140453149	C|G	ENSP00000418033	G24R	Un.S
rs121913364	140453134	T|C	ENSP00000418033	K29E	Path/2 L.Path/1
rs121913366	140453145	A|T	ENSP00000418033	L25Q	Path/1
rs121913366		A|C	ENSP00000418033	L25R	
rs121913375	140453139	G|A	ENSP00000418033	T27I	Path/1
rs397507483	140453148	C|A	ENSP00000418033	G24Y	Path/3
rs397507484	140453133	T|A	ENSP00000418033	K29I	Path/1 L.Path/1
rs121913225	140453151	A|G	ENSP00000418033	F23S	—
rs121913337	140453153	A|T	ENSP00000418033	D22E	—
rs121913362	140453159	T|C	ENSP00000418033	I20M	—
rs121913365	140453132	T|G	ENSP00000418033	K29N	—
rs180177042	140449165	A|T	ENSP00000418033	D66E	Path/2
rs199927105	140482930	G|T	ENSP00000419060	P10H	—
rs121913357	140481403	C|T	ENSP00000419060	G77R	Path/3 L.Path/1 Un.S/0
rs180177033	140481420	A|C	ENSP00000419060	I71S	Path/1
rs397507473	140481405	A|G	ENSP00000419060	F76S	Path/2
rs121913371	140481478	G|A	ENSP00000419060	R52W	—
rs121913351	140481411	C|A	ENSP00000419060	G74V	Path/2
rs121913351		C|T	ENSP00000419060	G74E	
rs121913355	140481402	C|G	ENSP00000419060	G77A	Path/5
rs121913355		C|A	ENSP00000419060	G77V	
rs121913355		C|T	ENSP00000419060	G77E	
rs180177036	140477853	C|G	ENSP00000419060	L93F	Path/2
rs180177037	140477813	T|C	ENSP00000419060	K107E	Path/2
rs180177038	140477807	C|T	ENSP00000419060	E109K	Path/3 L.Path/1
rs180177039	140477806	T|C	ENSP00000419060	E109G	Path/2 L.Path/1
rs397507479	140476811	C|T	ENSP00000419060	C140Y	Path/1 Un.S/1
rs180177040	140453987	T|G	ENSP00000419060	N189H	Path/2
rs180177040		T|C	ENSP00000419060	N189D	
rs397507481	140454006	G|C	ENSP00000419060	H182Q	Path/1
rs121913341	140453150	A|C	ENSP00000419060	F203L	Path/2 L.Path/1
rs121913361	140453149	C|G	ENSP00000419060	G204R	Un.S/0
rs121913364	140453134	T|C	ENSP00000419060	K209E	Path/5
rs121913366	140453145	A|T	ENSP00000419060	L205Q	Path/1
rs121913366		A|C	ENSP00000419060	L205R	
rs121913370	140453193	T|C	ENSP00000419060	N189S	L.Path/1
rs397507483	140453148	C|A	ENSP00000419060	G204V	Path/3
rs121913225	140453151	A|G	ENSP00000419060	F203S	—
rs121913337	140453153	A|T	ENSP00000419060	D202E	—
rs121913362	140453159	T|C	ENSP00000419060	I200M	—
rs372569965	140453127	C|T	ENSP00000419060	R211Q	—
rs180177042	140449165	A|T	ENSP00000419060	D246E	Path/2
rs397507485	14043972	C|T	ENSP00000419060	R279Q	Path/1
rs397507486	140439613	T|C	ENSP00000419060	Q317R	Path/1
rs397507487	140434543	G|A	ENSP00000419060	R327C	Path/1
rs200490285	140434452	G|T	ENSP00000419060	A357D	—
rs397507476	140477811	T|G	ENSP00000419060	K107N	Path/1
rs150050723	140534634	T|G	ENSP00000420119	Q78H	—
rs180177032	140481423	C|A	ENSP00000419060	R70I	Path/1
rs121913378	140453137	C|T	ENSP00000288602	V600M	L.Path/1
rs121913369	140453146	G|C	ENSP00000288602	L597V	Path/4
rs121913369		G|C	ENSP00000419060	L205V	
rs121913378	140453137	C|T	ENSP00000419060	V208M	L.Path/1
rs397507481	140454006	G|C	ENSP00000418033	H2Q	Path/1

SNP ID refers to dbSNP. Ch7: location within chromosome number seven (assembly GRCh37/hg19). Clin/sig: clinical significance refers to ClinVar database; significant results could be one of the following: Path: pathogenic, benign; L.Path: likely pathogenic, or/and Un.S: unsignificant. Number after significant results refers to number of diseases that are associated with this SNP.

**Table 3:** Functional SNPs predicted by triple servers.

Protein ID	Amino acid change	SIFT prediction	Score	PolyPhen-2 prediction	Score	SNAP2	Score
ENSP00000288602	Q93H	Damaging	0	Probably damaging	0.974	Effect	15
ENSP00000288602	A246P	Damaging	0	Probably damaging	0.999	Effect	73
ENSP00000288602	T241M	Damaging	0	Probably damaging	1	Effect	72
ENSP00000288602	T241R	Damaging	0	Probably damaging	1	Effect	73
ENSP00000288602	T241P	Damaging	0	Probably damaging	1	Effect	86
ENSP00000288602	L245F	Damaging	0	Probably damaging	0.999	Effect	64
ENSP00000288602	F247S	Damaging	0.01	Possibly damaging	0.443	Effect	77
ENSP00000288602	F247L	Damaging	0	Probably damaging	0.987	Effect	70
ENSP00000288602	P367L	Damaging	0.01	Probably damaging	0.999	Effect	36
ENSP00000288602	P402H	Damaging	0	Probably damaging	0.948	Effect	29
ENSP00000288602	G464A	Damaging	0	Probably damaging	0.999	Effect	50
ENSP00000288602	G464V	Damaging	0	Probably damaging	1	Effect	70
ENSP00000288602	G464E	Damaging	0	Probably damaging	1	Effect	84
ENSP00000288602	G466R	Damaging	0	Probably damaging	0.996	Effect	95
ENSP00000288602	G469R	Damaging	0	Probably damaging	1	Effect	90
ENSP00000288602	V471F	Damaging	0	Probably damaging	0.954	Effect	78
ENSP00000288602	V471I	Damaging	0	Possibly damaging	0.22	Effect	37
ENSP00000288602	I463S	Damaging	0	Possibly damaging	0.714	Effect	75
ENSP00000288602	F468S	Damaging	0	Possibly damaging	0.67	Effect	82
ENSP00000288602	G464R	Damaging	0	Probably damaging	1	Effect	79
ENSP00000288602	R444W	Damaging	0	Probably damaging	0.999	Effect	67
ENSP00000288602	G466A	Damaging	0	Probably damaging	0.97	Effect	87
ENSP00000288602	G466V	Damaging	0	Probably damaging	0.983	Effect	93
ENSP00000288602	G466E	Damaging	0	Probably damaging	0.996	Effect	94
ENSP00000288602	G469A	Damaging	0	Probably damaging	0.969	Effect	73
ENSP00000288602	G469V	Damaging	0	Probably damaging	1	Effect	89
ENSP00000288602	G469E	Damaging	0	Probably damaging	1	Effect	93
ENSP00000288602	L485F	Damaging	0	Probably damaging	0.999	Effect	5
ENSP00000288602	E501K	Damaging	0.01	Probably damaging	0.997	Effect	84
ENSP00000288602	E501G	Damaging	0	Probably damaging	1	Effect	81
ENSP00000288602	K483Q	Damaging	0	Probably damaging	0.999	Effect	79
ENSP00000288602	L485S	Damaging	0	Probably damaging	0.999	Effect	42
ENSP00000288602	K499N	Damaging	0	Probably damaging	0.884	Effect	58
ENSP00000288602	L505F	Damaging	0.01	Possibly damaging	0.698	Effect	41
ENSP00000288602	P490S	Damaging	0	Possibly damaging	0.494	Effect	31
ENSP00000288602	G534R	Damaging	0	Probably damaging	1	Effect	34
ENSP00000288602	C532Y	Damaging	0	Probably damaging	1	Effect	75
ENSP00000288602	N581H	Damaging	0	Probably damaging	1	Effect	73
ENSP00000288602	N581D	Damaging	0.05	Possibly damaging	0.503	Effect	76
ENSP00000288602	H574Q	Damaging	0	Probably damaging	0.999	Effect	89
ENSP00000288602	F595L	Damaging	0	Probably damaging	1	Effect	92
ENSP00000288602	G596R	Damaging	0	Probably damaging	1	Effect	95
ENSP00000288602	K601E	Damaging	0	Probably damaging	0.997	Effect	58
ENSP00000288602	L597Q	Damaging	0	Probably damaging	1	Effect	71
ENSP00000288602	L597R	Damaging	0	Probably damaging	0.999	Effect	81
ENSP00000288602	N581S	Damaging	0.04	Possibly damaging	0.517	Effect	56
ENSP00000288602	T599I	Damaging	0	Probably damaging	0.997	Effect	62
ENSP00000288602	G596V	Damaging	0	Probably damaging	1	Effect	92
ENSP00000288602	K601I	Damaging	0	Probably damaging	0.986	Effect	18
ENSP00000288602	F595S	Damaging	0	Probably damaging	1	Effect	95
ENSP00000288602	D594E	Damaging	0	Probably damaging	0.999	Effect	90
ENSP00000288602	I592M	Damaging	0	Probably damaging	0.997	Effect	55
ENSP00000288602	K601N	Damaging	0	Probably damaging	0.939	Effect	49
ENSP00000288602	R603Q	Damaging	0.03	Probably damaging	0.971	Effect	37
ENSP00000288602	D638E	Damaging	0	Probably damaging	1	Effect	91
ENSP00000420119	Q78H	Damaging	0	Probably damaging	0.948	Effect	41
ENSP00000288602	R671Q	Damaging	0	Probably damaging	0.996	Effect	28
ENSP00000288602	Q709R	Damaging	0.01	Possibly damaging	0.776	Effect	45
ENSP00000288602	L692S	Damaging	0	Probably damaging	0.999	Effect	30
ENSP00000288602	R719C	Damaging	0	Probably damaging	0.996	Effect	34
ENSP00000288602	A749D	Damaging	0.03	Possibly damaging	0.819	Effect	47
ENSP00000288602	R719L	Damaging	0	Possibly damaging	0.551	Effect	45
ENSP00000418033	N9H	Damaging	0	Probably damaging	0.98	Effect	66
ENSP00000418033	F23L	Damaging	0	Probably damaging	0.993	Effect	69
ENSP00000418033	G24R	Damaging	0	Probably damaging	1	Effect	90
ENSP00000418033	K29E	Damaging	0.04	Probably damaging	0.909	Effect	41
ENSP00000418033	L25Q	Damaging	0	Probably damaging	0.996	Effect	42
ENSP00000418033	L25R	Damaging	0	Probably damaging	0.996	Effect	61
ENSP00000418033	T27I	Damaging	0.01	Probably damaging	0.996	Effect	64
ENSP00000418033	G24Y	Damaging	0	Probably damaging	1	Effect	79
ENSP00000418033	K29I	Damaging	0	Probably damaging	0.907	Effect	8
ENSP00000418033	F23S	Damaging	0	Probably damaging	0.996	Effect	87
ENSP00000418033	D22E	Damaging	0	Probably damaging	0.997	Effect	80
ENSP00000418033	I20M	Damaging	0	Probably damaging	0.945	Effect	28
ENSP00000418033	K29N	Damaging	0	Probably damaging	0.949	Effect	35
ENSP00000418033	D66E	Damaging	0	Probably damaging	0.997	Effect	93
ENSP00000419060	P10H	Damaging	0	Probably damaging	0.976	Effect	6
ENSP00000419060	G77R	Damaging	0	Probably damaging	0.962	Effect	89
ENSP00000419060	I71S	Damaging	0	Probably damaging	0.999	Effect	86
ENSP00000419060	F76S	Damaging	0	Probably damaging	0.999	Effect	64
ENSP00000419060	R52W	Damaging	0	Probably damaging	0.976	Effect	55
ENSP00000419060	G74V	Damaging	0	Possibly damaging	0.351	Effect	81
ENSP00000419060	G74E	Damaging	0	Possibly damaging	0.51	Effect	87
ENSP00000419060	G77A	Damaging	0	Possibly damaging	0.344	Effect	52
ENSP00000419060	G77V	Damaging	0	Possibly damaging	0.276	Effect	82
ENSP00000419060	G77E	Damaging	0	Probably damaging	0.883	Effect	89
ENSP00000419060	L93F	Damaging	0	Probably damaging	0.919	Effect	6
ENSP00000419060	K107E	Damaging	0	Probably damaging	0.989	Effect	50
ENSP00000419060	E109K	Damaging	0.01	Probably damaging	0.992	Effect	91
ENSP00000419060	E109G	Damaging	0	Probably damaging	0.998	Effect	90
ENSP00000419060	C140Y	Damaging	0	Probably damaging	0.932	Effect	81
ENSP00000419060	N189H	Damaging	0	Probably damaging	0.996	Effect	66
ENSP00000419060	N189D	Damaging	0.03	Probably damaging	0.998	Effect	66
ENSP00000419060	H182Q	Damaging	0	Probably damaging	0.999	Effect	72
ENSP00000419060	F203L	Damaging	0	Probably damaging	1	Effect	89
ENSP00000419060	G204R	Damaging	0	Probably damaging	0.999	Effect	90
ENSP00000419060	K209E	Damaging	0	Possibly damaging	0.477	Effect	51
ENSP00000419060	L205Q	Damaging	0	Probably damaging	1	Effect	56
ENSP00000419060	L205R	Damaging	0	Probably damaging	1	Effect	75
ENSP00000419060	N189S	Damaging	0.03	Probably damaging	0.989	Effect	43
ENSP00000419060	G204V	Damaging	0	Possibly damaging	0.242	Effect	87
ENSP00000419060	F203S	Damaging	0	Probably damaging	1	Effect	94
ENSP00000419060	D202E	Damaging	0	Probably damaging	1	Effect	87
ENSP00000419060	I200M	Damaging	0	Probably damaging	1	Effect	32
ENSP00000419060	R211Q	Damaging	0.05	Probably damaging	0.979	Effect	8
ENSP00000419060	D246E	Damaging	0	Probably damaging	0.998	Effect	96
ENSP00000419060	R279Q	Damaging	0.02	Probably damaging	1	Effect	56
ENSP00000419060	Q317R	Damaging	0.03	Probably damaging	0.975	Effect	2
ENSP00000419060	R327C	Damaging	0	Possibly damaging	0.291	Effect	43
ENSP00000419060	A357D	Damaging	0.03	Possibly damaging	0.507	Effect	22
ENSP00000419060	K107N	Damaging	0	Probably damaging	0.994	Effect	32

**Table 4:** Functional SNPs predicted by double tools.

Protein ID	Amino acid change	SIFT prediction	Score	PolyPhen-2 prediction	Score	SNAP2 prediction	Score
ENSP00000419060	R70I	Damaging	0	Benign	0.311	Effect	34
ENSP00000288602	V600M	Damaging	0	Probably damaging	0.99	Neutral	−20
ENSP00000288602	L597V	Damaging	0	Probably damaging	0.862	Neutral	0
ENSP00000419060	L205V	Damaging	0	Probably damaging	0.995	Neutral	−13
ENSP00000419060	V208M	Damaging	0.03	Probably damaging	0.996	Neutral	−28
ENSP00000418033	H2Q	Damaging	0	Probably damaging	0.998	Neutral	−13

**Table 5:** Predicted results of I-Mutant software.

Protein ID	Amino acid position	WT	MT	PH	Temperature (°C)	SVM2 prediction effect	DDG value prediction kcal/mol	RI
ENSP00000288602	466	G	R	7.0	25	Increase	−0.24	4
ENSP00000288602	469	G	R	7.0	25	Increase	−0.01	4
ENSP00000288602	466	G	V	7.0	25	Increase	−0.08	2
ENSP00000288602	466	G	E	7.0	25	Decrease	−0.86	6
ENSP00000288602	469	G	E	7.0	25	Increase	−0.18	5
ENSP00000288602	595	F	L	7.0	25	Decrease	−0.73	8
ENSP00000288602	596	G	R	7.0	25	Decrease	−0.24	1
ENSP00000288602	596	G	V	7.0	25	Decrease	−0.20	2
ENSP00000288602	595	F	S	7.0	25	Decrease	−1.27	8
ENSP00000288602	638	D	E	7.0	25	Decrease	−1.68	7
ENSP00000418033	24	G	R	7.0	25	Decrease	−1.88	8
ENSP00000418033	66	D	E	7.0	25	Increase	−0.31	3
ENSP00000419060	109	E	K	7.0	25	Decrease	−0.69	8
ENSP00000419060	203	F	S	7.0	25	Decrease	−1.27	8
ENSP00000419060	246	D	E	7.0	25	Decrease	−1.68	7

WT: wild type amino acid. MT: mutant type amino acid. DDG: delta DG (units of free energy) (DDG < 0: decreased stability, DDG > 0: increased stability). RI: reliability index.

**Table 6:** Predicted results of functional 3′ UTR SNPs/Indels.

dbSNP ID	Variant type	Ancestral allele	Allele	miR ID	Conservation	miRSite	Function class	Context + score change
rs114105685	SNP	G	A	hsa-miR-30a-5p	3	gTGTTT**A**CAggtg	C	−0.083
				hsa-miR-30b-5p	3	gTGTTT**A**CAggtg	C	−0.104
				hsa-miR-30c-5p	3	gTGTTT**A**CAggtg	C	−0.104
				hsa-miR-30d-5p	3	gTGTTT**A**CAggtg	C	−0.083
				hsa-miR-30e-5p	3	gTGTTT**A**CAggtg	C	−0.083
				hsa-miR-3607-3p	4	gtgTTT**A**CAGgtg	C	No change

rs184804021	SNP	T	T	hsa-miR-3908	4	tagaCA**T**TGCTAa	D	−0.344
				hsa-miR-3942-5p	5	tagacA**T**TGCTAa	D	−0.102
				hsa-miR-4703-5p	5	tagacA**T**TGCTAa	D	−0.099
				hsa-miR-4766-3p	5	tagacA**T**TGCTAA	D	−0.256
			C	hsa-miR-374b-3p	5	tagaca**C**TGCTAA	C	−0.253
				hsa-miR-4274	5	tagacA**C**TGCTAa	C	−0.161
				hsa-miR-4519	4	tagaCA**C**TGCTAa	C	−0.375
				hsa-miR-4524a-5p	5	tagaca**C**TGCTAA	C	−0.139
				hsa-miR-4524b-5p	5	tagaca**C**TGCTAA	C	−0.139
				hsa-miR-6499-3p	7	tagACA**C**TGCtaa	C	−0.222
				hsa-miR-6733-3p	5	taGACA**C**TGctaa	C	−0.275

rs140083479	SNP	A	A	hsa-miR-3908	4	atagaC**A**TTGCTA	D	−0.344
				hsa-miR-3942-5p	5	atagac**A**TTGCTA	D	−0.102
				hsa-miR-4703-5p	5	atagac**A**TTGCTA	D	−0.099
				hsa-miR-4766-3p	5	atagac**A**TTGCTA	D	−0.158
				hsa-miR-4796-5p	3	ATAGAC**A**ttgcta	D	−0.131
			T	hsa-miR-548p	5	atagac**T**TTGCTA	C	−0.07

rs200393520	Indel	—	—	hsa-miR-4311	2	acTCTC**T**TTtttt	O	0.004
			T					

rs143647707	SNP	A	A	hsa-miR-5580-3p	3	gaaCAT**A**TGTttg	D	−0.16
			T	hsa-miR-7-1-3p	4	gaacAT**T**TGTTtg	C	−0.053
				hsa-miR-7-2-3p	4	gaacAT**T**TGTTtg	C	−0.053

rs202148822	SNP	G	G	hsa-miR-1976	3	tCAGGA**G**Agtagc	D	−0.147
				hsa-miR-6845-3p	3	tcAGGA**G**AGtagc	D	−0.193

Conservation: occurrence of the miRNA site in other vertebrate genomes in addition to the query genome. miRSite: sequence context of the miRNA site: bases complementary to the seed region are in capital letters and SNPs are highlighted in bold font. Function class: D: the derived allele disrupts a conserved miRNA site (ancestral allele with support > 2); C: the derived allele creates a new miRNA site; O: the ancestral allele can not be determined. Context score: negative increase = increase in SNP functionality.

**Table 7:** 5′ UTR SNPs within TF binding sites.

SNP ID	Transcription factor predicted	Prediction
rs71645935	Pax-5, p53	No effect
rs71645936	FOXP3	No effect
rs397507453	None	—
rs762432076	None	—
rs769116177	None	—

**Table 8:** Polymorphisms and impact on splice sites.

Polymorphism ID	Type of splice site	Prediction
rs199910929 (SNP)	5′ splice site	(i) Alteration of an exonic ESE site (ii) Potential alteration of splicing
rs776683449 (SNP)	3′ splice site	No significant splicing motif alteration detected This mutation has probably no impact on splicing
rs775598011 (Indel)	3′ splice site	(i) Alteration of an exonic ESE site (ii) Potential alteration of splicing
